# KHSRP promotes cancer stem cell maintenance, tumorigenesis, and suppresses anti-tumor immunity in gastric cancer

**DOI:** 10.32604/or.2024.058273

**Published:** 2025-01-16

**Authors:** YARU DU, ZHIHUI PEI, SHUQIN HU, CHUANWEN LIAO, SHUHAO LIU

**Affiliations:** 1 Gastrointestinal Hernia Surgery, Jiangxi Provincial People’s Hospital, The First Affiliated Hospital of Nanchang Medical College, Nanchang, 330006, China; 2 Scientific Research Center, The Seventh Affiliated Hospital of Sun Yat-sen University, Shenzhen, 518107, China; 3 Jiangxi Medical College, Nanchang University, Nanchang, 330006, China; 4 Organ Transplantation Center, Jiangxi Provincial People’s Hospital, The First Affiliated Hospital of Nanchang Medical College, Nanchang, 330006, China

**Keywords:** KH-type splicing regulatory protein (KHSRP), Gastric cancer (GC), Cancer stem cell, Tumorigenesis

## Abstract

**Objectives:**

KH-type splicing regulatory protein (KHSRP) is an RNA-binding protein involved in several cellular processes, including nuclear splicing, mRNA localization, and cytoplasmic degradation. While KHSRP’s role has been studied in other cancers, its specific involvement in gastric cancer remains poorly understood. This study aims to explore KHSRP expression in gastric cancer and its potential effects on tumor progression and immune response.

**Methods:**

KHSRP expression in gastric cancer tissues and normal tissues was analyzed using data from The Cancer Genome Atlas (TCGA) database. The correlation between KHSRP expression, patient survival, and immune response was also assessed. Immunohistochemistry was performed to evaluate KHSRP expression in gastric cancer tissues. Gain- and loss-of-function experiments were conducted to assess KHSRP’s effects on gastric cancer cell proliferation, stemness, and migration. Furthermore, the impact of KHSRP silencing on tumor volume and immune cell infiltration was evaluated in a C3H/He mouse xenograft model.

**Results:**

KHSRP was found to be overexpressed in gastric cancer tissues compared to normal tissues, with a positive correlation to tumor stage and a negative correlation with patient prognosis. Functional assays revealed that KHSRP promotes gastric cancer cell proliferation, enhances cancer stem cell properties, and increases migratory capabilities *in vitro*. *In vivo*, KHSRP silencing led to a significant reduction in tumor volume and increased immune cell infiltration in the mouse xenograft model.

**Conclusions:**

KHSRP acts as an oncogene in gastric cancer by promoting tumorigenesis and suppressing anti-tumor immune responses. Its overexpression is associated with poor prognosis, making KHSRP a potential prognostic marker and therapeutic target in gastric cancer.

## Introduction

Gastric cancer (GC) is a leading malignancy worldwide, ranking as the fifth most common cancer and the fourth leading cause of cancer-related deaths globally [1,2]. While the incidence of gastric cancer varies by region, there is a troubling increase in cases among individuals under 50 years old, with a reported rise of 25% in low-risk countries and 40% in high-risk countries [3]. Besides *Helicobacter pylori* infection, the development of gastric cancer is associated with genetic predispositions and lifestyle factors such as alcohol consumption and smoking [4,5]. Unfortunately, the lack of clear clinical symptoms often results in late-stage diagnosis for many patients, contributing to a poor prognosis [6,7]. The prognosis for advanced gastric cancer patients remains grim, with a 5-year survival rate of less than 10%. For approximately half of the patients eligible for surgery, adjuvant chemotherapy or chemoradiotherapy is the standard post-surgical treatment [8]. However, despite surgical resection, approximately 60% of patients experience local recurrence or distant metastasis [9]. Moreover, many patients with disseminated tumors are ineligible for surgery. Peritoneal metastasis is particularly common in gastric cancer, affecting nearly one-third of patients at the time of diagnosis [10]. Systemic chemotherapy remains the primary treatment for metastatic GC, with a median overall survival (OS) of approximately 12 months for those receiving conventional chemotherapy [11]. Thus, advanced diagnostic techniques and therapies are essential for better characterizing the molecular features of gastric cancer patients and identifying potential new therapeutic targets [12–14]. Understanding the etiology and mechanisms underlying the development of gastric cancer is crucial. This knowledge could pave the way for the identification of new diagnostic and prognostic molecular markers, as well as novel molecular targets of clinical significance, ultimately enhancing treatment outcomes and improving overall survival.

Emerging evidence suggests that tumor cells are highly heterogeneous and adaptable. Within tumors, a small subset of cells, known as cancer stem cells (CSCs) or tumor-initiating cells, possess unlimited proliferative potential and the ability to reinitiate tumor formation. These CSCs maintain a stable presence in tumors through their self-renewal and differentiation capabilities [15–17]. CSCs are recognized as key drivers of tumorigenesis, drug resistance, recurrence, and metastasis, making them a significant factor in therapeutic failure in cancer treatment [18]. In gastric cancer, metastasis, recurrence, and resistance to chemotherapy are the primary contributors to patient mortality. According to the cancer stem cell theory, CSCs are central to these processes, making them crucial targets for gastric cancer treatment [19,20]. For gastric cancer stem cells (GCSCs), genes and non-coding RNAs play key regulatory roles. Studies have demonstrated that various drugs can modulate the stemness of gastric cancer by targeting these regulatory elements [21–23]. Therefore, the strategic targeting and eradication of CSCs present new opportunities for improving outcomes in metastatic gastric cancer and could open new avenues for clinical treatment.

The KH-type splicing regulatory protein (KHSRP), also known as FUBP2 (Far Upstream Element-Binding Protein 2) [24], is a multifunctional RNA-binding protein that plays a crucial role in various aspects of RNA metabolism, including splicing, stability, and translation [25]. Recent research has underscored the role of KHSRP in cancer, indicating its potential influence on tumor progression, metastasis, and treatment resistance. KHSRP promotes the degradation of specific mRNAs by binding to AU-rich elements (AREs) in their 3′ untranslated regions (UTRs). This activity can affect the expression of genes involved in cancer progression, including those related to cell cycle regulation, apoptosis, and angiogenesis [26,27]. KHSRP can modulate the expression of oncogenes and tumor suppressor genes by regulating mRNA stability. For example, KHSRP has been shown to downregulate the mRNAs of tumor suppressors, thereby promoting tumor growth and proliferation [28,29]. KHSRP may also contribute to the maintenance of CSCs, which are thought to drive tumor initiation, therapy resistance, and recurrence [26]. By regulating the stability of mRNAs associated with stemness factors such as SOX2, OCT4, and Nanog, KHSRP could support the self-renewal and survival of CSCs [30–32]. KHSRP has been associated with chemotherapy resistance in certain cancers. By modulating the expression of genes involved in drug metabolism, DNA repair, and apoptosis, KHSRP can enhance the resistance of cancer cells to chemotherapy, contributing to treatment failure [33,34]. Given its involvement in multiple cancer-related processes, KHSRP is being investigated as a potential therapeutic target. Inhibiting KHSRP function could restore the expression of tumor suppressor genes, reduce epithelial-mesenchymal transition (EMT) and metastasis, and sensitize cancer cells to chemotherapy [35–37].

KHSRP plays a critical role in cancer biology, impacting various aspects of tumor progression, metastasis, and treatment resistance. Its diverse functions in RNA metabolism position it as a potential target for novel cancer therapies, particularly in tumors where KHSRP expression is dysregulated. However, its role and underlying mechanisms in gastric cancer remain poorly understood. This study aims to investigate the role of KHSRP in gastric cancer stem cells and tumor metastasis, further elucidating its contribution to gastric cancer growth and uncovering its potential molecular mechanisms.

## Materials and Methods

### Cell lines and cell culture

The HEK-293T (293T) (Pricella, CL-0005, Wuhan, China) and human GC cell lines HGC-27 and MKN-45 were obtained from the Cell Bank of Type Culture Collection of the Chinese Academy of Sciences (Shanghai, China). The GC cell lines were cultured in Roswell Park Memorial Institute (RPMI) 1640 medium (Gibco, 11875119, Shanghai, China) supplemented with 10% fetal bovine serum (FBS) (Mikxlife, MK1123, Shenzhen, China), while the 293T cells were maintained in Dulbecco’s Modified Eagle’s Medium (DMEM, high glucose) (Gibco, 11965) with 10% FBS. All cells were kept in a humidified incubator at 37°C with 5% carbon dioxide. All cells used in this study were free of mycoplasma contamination.

### Plasmid construction and transfection

The human KHSRP overexpression plasmid (pLV3-CMV-3xFlag) and knockdown plasmid (pLKO.1-puro) were purchased from Novopro (V015125, Shanghai, China) and TranSheep (TV00431, Shanghai, China), respectively. For lentivirus production and subsequent infection, the lentiviral target plasmid, packaging plasmid pSPAX2 (Packaging Plasmid) (Addgene, 12260, Beijing, China), and envelope plasmid PDM2.0g (Envelope Plasmid) (Addgene, 12259) were co-transfected into 293T cells using Lipofectamine 2000 (Invitrogen, 11668019, Shanghai, China). Viral supernatants were collected at 48 and 72 h post-transfection, filtered through a 0.45 µm sterile filter (MerckMillipore, SLHVR33RB, Shanghai, China), and used to infect gastric cancer cells. Hexadimethrine bromide (Polybrene) (Santa Cruz, sc-134220, Shanghai, China) was added at a concentration of 5 µg/mL during infection. After 48 h, cells were selected with 1 µg/mL puromycin (Solarbio, P8230, Shanghai, China) for 72 h to establish overexpression and knockdown cell lines. The sh-β-catenin cell line was constructed using the same plasmids and method. The shRNA sequences for KHSRP and β-catenin as follows: (1) Control-shRNA: TGTTATGTTTACACAGGCCTTTTTT; KHSRP-shRNA-1: AGCAATCTTCTCGGGTTTTTT; KHSRP-shRNA-2: TGTGAGTAGTAGGCGTTTTTT; KHSRP-shRNA-3: TTCTGTCATTGAAGTCTTTTTT (2) Control-shRNA: ATGGTAGCGTACACTTATGAT; β-catenin-shRNA: GCTCCTGTCTAATGCTTAGTT.

### Western blotting

Proteins from cells and tissues were extracted using Radio-Immunoprecipitation Assay Buffer (RIPA buffer) (Beyotime, P0013B, Shanghai, China) or Tissue Protein Extraction Reagent (ThermoFisher, 78510, Shanghai, China) supplemented with 1% protease and phosphatase inhibitors. Protein concentrations were determined using the Pierce Bicinchoninic Acid Protein Assay Kit (ThermoFisher, 23227). The extracts were separated by Sodium Dodecyl Sulfate-Polyacrylamide Gel Electrophoresis (SDS-PAGE) and transferred onto Polyvinylidene Fluoride Membranes (PVDF membranes) (Millipore, IPVH00010). The membranes were blocked with Phosphate-Buffered Saline with Tween-20 (PBST buffer) (MerckMillipore, 524653) buffer containing 5% Bovine Serum Albumin (BSA) (BioFroxx, 4240GR100, Shanghai, China) at room temperature for 1 h, followed by incubation with the primary antibody overnight at 4°C. The membranes were then washed three times with PBST, 5 min each time, and incubated with the secondary antibody at room temperature for 1 h. The primary antibodies used are listed in Table S1. Immunoreactive bands were visualized using an Enhanced Chemiluminescence Kit (ECL kit) (Millipore, WBULP-100 ML).

### Cell proliferation

The proliferation of gastric cancer cells was evaluated using the Cell Counting Kit-8 (CCK8) (Abbkine, KTA1020, Wuhan, China). Briefly, following the overexpression or knockdown of KHSRP, the cells were seeded into 96-well plates at a density of approximately 5000 cells per well. Starting the day after seeding, CCK8 assays were conducted daily for 4 consecutive days. For each assay, 10 µL of CCK8 reagent was added to 100 µL of culture medium, and the cells were incubated in the dark at 37°C for 2 h. Absorbance was measured at 450 nm, and the results were analyzed statistically.

### Tumorsphere formation assay

Gastric cancer cells were dissociated into single-cell suspensions and seeded into ultra-low attachment plates (Corning, 3473, Shanghai, China) at a density of 2000–5000 cells per well in serum-free DMEM medium. The cells were cultured in a humidified incubator at 37°C with 5% carbon dioxide for 7–10 days without disturbing the plates. Tumorspheres were observed and imaged under a microscope (Mshot, MF52-N). Sphere size and number were quantified to assess self-renewal capacity.

### Transwell migration and invasion assays

Collect gastric cancer cells with KHSRP overexpression or knockdown, as well as control cells, and centrifuge them. Suspend the cells in serum-free RPMI 1640 medium. For the migration assay, seed 1 × 10^⁴^ viable cells in 200 µL of serum-free medium into the upper chamber of a Transwell plate with an 8 µm pore membrane (Corning, 354480). The lower chamber should contain 600 µL of culture medium with 20% FBS to facilitate cell migration through the membrane. For the invasion assay, seed 2 × 10^⁴^ viable cells onto a Matrigel-coated membrane (Corning, 354603). As in the migration assay, a medium containing serum in the lower chamber serves as a chemoattractant. After 48 h of incubation, wash the migrated cells with Phosphate-Buffered Saline (PBS) (Biosharp, BL302A, Hefei, China), fix them with 4% paraformaldehyde (PFA) (Biosharp, BL539A), and stain with 0.5% crystal violet (Beyotime, C0121). Carefully remove the non-migrated cells using a cotton swab. Randomly select three fields under an inverted microscope (Mshot, MF52-N) for cell counting and photography.

### Hematoxylin and eosin (H&E) staining assay

Tissue sections were deparaffinized by immersing them in dimethylbenzene (ThermoFisher, 1330-20-7) twice for 5 min each. Rehydration was performed through graded ethyl alcohols (ThermoFisher, 64-17-5) series (100%, 95%, 85%, and 75%), with each step lasting 10 min. The sections were then immersed in PBS to maintain hydration. Hematoxylin staining was carried out by incubating the sections in hematoxylin staining solution (Solarbio, G1120) for 5 min, followed by rinsing under running tap water to remove excess stain. Eosin staining (Solarbio, G1120) was performed by immersing the sections in an eosin staining solution for 2 min. Subsequently, the sections were dehydrated through a series of graded ethyl alcohols (75%, 85%, 95%, and 100%) for 10 min each, followed by two washes in dimethylbenzene, 5 min each, to complete the dehydration process. Finally, the mounting medium was applied, coverslips were placed, and the stained samples were visualized under a light microscope (Mshot, MF52-N).

### Immunohistochemistry (IHC) staining assay

For immunohistochemical staining, tumor tissues were fixed in 4% paraformaldehyde, sectioned, and deparaffinized. After rehydration through a graded alcohol series, sodium citrate-phosphate buffer (Biosharp, BL619A) was brought to a boil in a microwave oven, and the sections were immersed in the sodium citrate-phosphate buffer for antigen retrieval at high temperature for 20 min. The sections were then treated with 3% hydrogen peroxide (G-clone, CS7730, Beijing, China) for 10 min to block endogenous peroxidase activity. Following this, the sections were blocked with 5% BSA for 1 h and then incubated with the primary antibody (The primary antibodies used are listed in Table S1) overnight at 4°C. The next day, the sections were incubated with the secondary antibody at room temperature for 1 h, followed by 3,3′-Diaminobenzidine (DAB) (Maxim, DAB-2031, Fuzhou, China) staining. The stained sections were then observed and imaged under a microscope (Mshot, MF52-N) by three independent pathologists. The staining intensity was scored as follows: 0 = no staining, 1 = weak staining, 2 = moderate staining, 3 = strong staining. The frequency of positive cells was scored as 0 =<10%, 1 = 10%–25%, 2 = 25%–50%, 3 = 50%–75%, 4 =>75%. The final IHC score was calculated by multiplying the staining intensity by the frequency of positive cells.

### Immunofluorescence staining assay

For immunofluorescence staining, cells or tissue sections were fixed with 4% paraformaldehyde for 15–20 min at room temperature, followed by permeabilization with 0.1% Octylphenol Ethylene Oxide Condensate (Triton X-100) (G-clone, CS9013) for 10 min. To block non-specific binding, samples were incubated with 5% BSA for 1 h at room temperature. Primary antibodies (The primary antibodies used are listed in Table S1) (CD8, Santa Cruz, sc-1177) diluted in blocking buffer were applied, and the samples were incubated overnight at 4°C. After washing three times with PBS, fluorescent-labeled secondary antibodies (Dylight 649, Goat Anti-Mouse IgG, Abbkine, A23610, Wuhan, China) were added and incubated for 1 h at room temperature in the dark. Following additional PBS washes, the samples were mounted with DAPI-containing medium (Beyotime, P0131) and covered with coverslips. Fluorescent images were acquired using a fluorescence microscope (Leica, DMI8).

### Flow cytometry assay

For flow cytometry, approximately 1 × 10^6^ cells were harvested, washed with PBS, and blocked with 2% fetal bovine serum (FBS) at 4°C for 15 min. Cells were then incubated with fluorescent-conjugated antibodies (The primary antibodies used are listed in Table S1) (CD44, ThermoFisher, 12-0441-82,) in staining buffer at 4°C for 30 min in the dark, washed twice with staining buffer. Samples were analyzed on a flow cytometer (Beckman, cytoflex LX), and the data were processed using FlowJo software (Becton, Dickinson & Company, Flowjo, version 10.6.2).

### Human samples and tissue microarray

Gastric and normal tissue microarrays were purchased from Shanghai Outdo Biotech Co., Ltd. (Shanghai, China). Immunohistochemistry was employed to analyze KHSRP expression and its correlation with survival in 80 human gastric tumors and normal tissue samples.

### Xenograft tumor model of GC cells

Four-week-old female C3H/He mice (18 in total, each weighing approximately 25–30 g) were purchased from GemPharmatech Co., Ltd. and housed under specific pathogen-free (SPF) conditions at the Experimental Animal Center of Sun Yat-sen University. The study protocol was approved by Sun Yat-sen University Institutional Animal Care and Use Committee (SYSU-IACUC-2024-B1575). After a one-week acclimatization period, the mice were subcutaneously injected with 80 μL of sterile PBS containing a suspension of 2 × 10^⁶^ GC cells/mL. Body weight and tumor volume were monitored, and two weeks later, all mice were humanely euthanized by cervical dislocation. Tumor tissues were then harvested for immunohistochemistry and immunofluorescence analysis.

## RT-qPCR Analysis

Total RNA was extracted from cells using the Tissue Total RNA Isolation Kit (Vazyme, RC112-01, Nanjing, China), according to the manufacturer’s instructions. The RNA concentration and purity were determined using a NanoDrop spectrophotometer (Thermo Fisher, 840-317400). For reverse transcription, 1 µg of total RNA was transcribed into complementary DNA (cDNA) using the HiScript IV All-in-One Ultra RT SuperMix for qPCR (Vazyme, R433-01). The cDNA was diluted 1:10 and stored at −20°C until use.

Quantitative PCR was performed using SYBR Green Master Mix (Vazyme, Q712), on a Real-Time PCR System (Bio-rad, 12011319, Guangzhou, China). The following primers were used for amplification: KHSRP-Forward primer: CCACAGCAGGACTACACGAA; KHSRP-Reverse primer: GGGTCTGTCCGTAGTAAGCG; GAPDH-Forward primer: AAATTCCATGGCACCGTCAA; GAPDH-Reverse primer: GCATCGCCCCACTTGATTTT. PCR conditions were as follows: an initial denaturation step at 95°C for 10 min, followed by 40 cycles of denaturation at 95°C for 15 s, annealing at 60°C for 30 s, and extension at 72°C for 30 s. A dissociation curve analysis was performed after each PCR run to ensure the specificity of the amplification. The expression levels of target genes were normalized to GAPDH.

### Statistical analysis

All *in vitro* experiments were independently repeated at least three times. Data are presented as mean ± standard deviation (SD) from at least three independent experiments. Statistical analysis was performed using GraphPad Prism 5 software (GraphPad, Beijing, China). A Student’s *t*-test was used for comparisons between two groups, and one-way ANOVA was applied for comparisons among three or more groups. A *p*-value of <0.05, <0.01, or <0.001 was considered statistically significant and denoted by *, **, and ***, respectively. A *p*-value ≥ 0.05 was considered not statistically significant and marked as NS.

## Results

### The expression of KHSRP in gastric cancer and its relationship with the clinical characteristics

**Figure 1:**
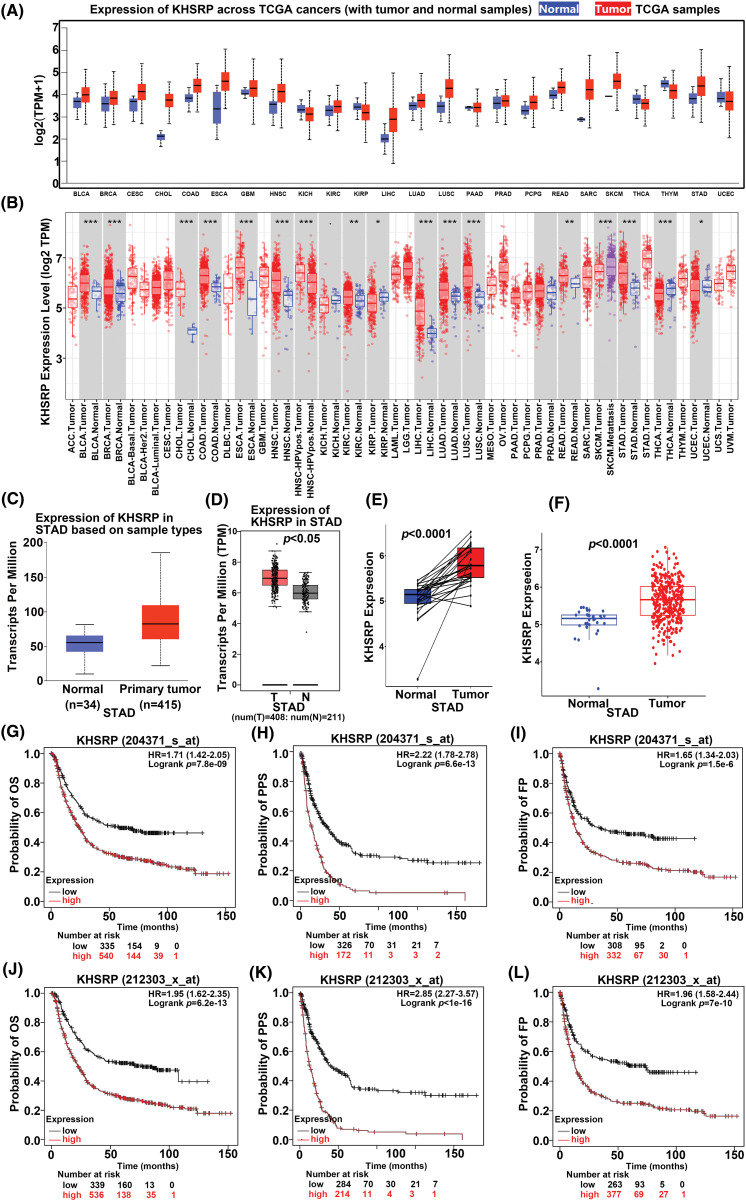
The expression KHSRP in gastric cancer and its relationship with the clinical characteristics. (A and B) KHSRP is highly expressed in various tumors, including gastric cancer. (C) KHSRP mRNA expression levels in the TCGA-STAD dataset. (D–F) KHSRP expression in gastric cancer tissues *vs*. adjacent non-cancerous tissues, as shown in the GEPIA database. (G–L) Kaplan-Meier analysis of the correlation between KHSRP expression and overall survival, relapse-free survival, and progression-free survival in STAD patients. OS: Overall survival; FP: First progression; PPS: Post progression survival. *p < 0.05, **p < 0.01, ***p < 0.001.

Initially, KHSRP expression across various cancers was analyzed using a database (Fig. 1A–B), revealing elevated levels in multiple tumor types, including Bladder Urothelial Carcinoma (BLCA), Breast Invasive Carcinoma (BRCA), Cervical Squamous Cell Carcinoma and Endocervical Adenocarcinoma (CESE), Cholangiocarcinoma (CHOL), Esophageal Carcinoma (ESCA), Head and Neck Squamous Cell Carcinoma (HNSC), Kidney Renal Clear Cell Carcinoma (KIRC), Lung Squamous Cell Carcinoma (LUSC), Pheochromocytoma and Paraganglioma (PCPG), Skin Cutaneous Melanoma (SKCM), Stomach Adenocarcinoma (STAD), Colon Adenocarcinoma (COAD), Glioblastoma Multiforme (GBM), Liver Hepatocellular Carcinoma (LIHC), Lung Adenocarcinoma (LUAD), Prostate Adenocarcinoma (PRAD), and Sarcoma (SARC). Notably, KHSRP expression was significantly elevated in stomach adenocarcinoma (STAD). Data from the TCGA database showed that KHSRP expression was higher in STAD tumors (415 cases) compared to normal tissues (34 cases) (Fig. 1C). This finding was further corroborated by analysis using the GEPIA database, which confirmed increased KHSRP expression in gastric cancer tissues (Fig. 1D–F). Additionally, KHSRP expression was particularly high in poorly differentiated gastric tumors (Supplementary Fig. 1B). Kaplan-Meier survival analysis demonstrated that patients with elevated KHSRP expression had a lower overall survival rate (Fig. 1G–L). In summary, these results suggest that KHSRP is significantly overexpressed in gastric cancer and is associated with reduced survival, implicating its role in the progression of the malignant phenotype in gastric cancer.

### KHSRP is significantly upregulated in gastric cancer tissues and is highly correlated with the malignancy of the tumor.

**Figure 2:**
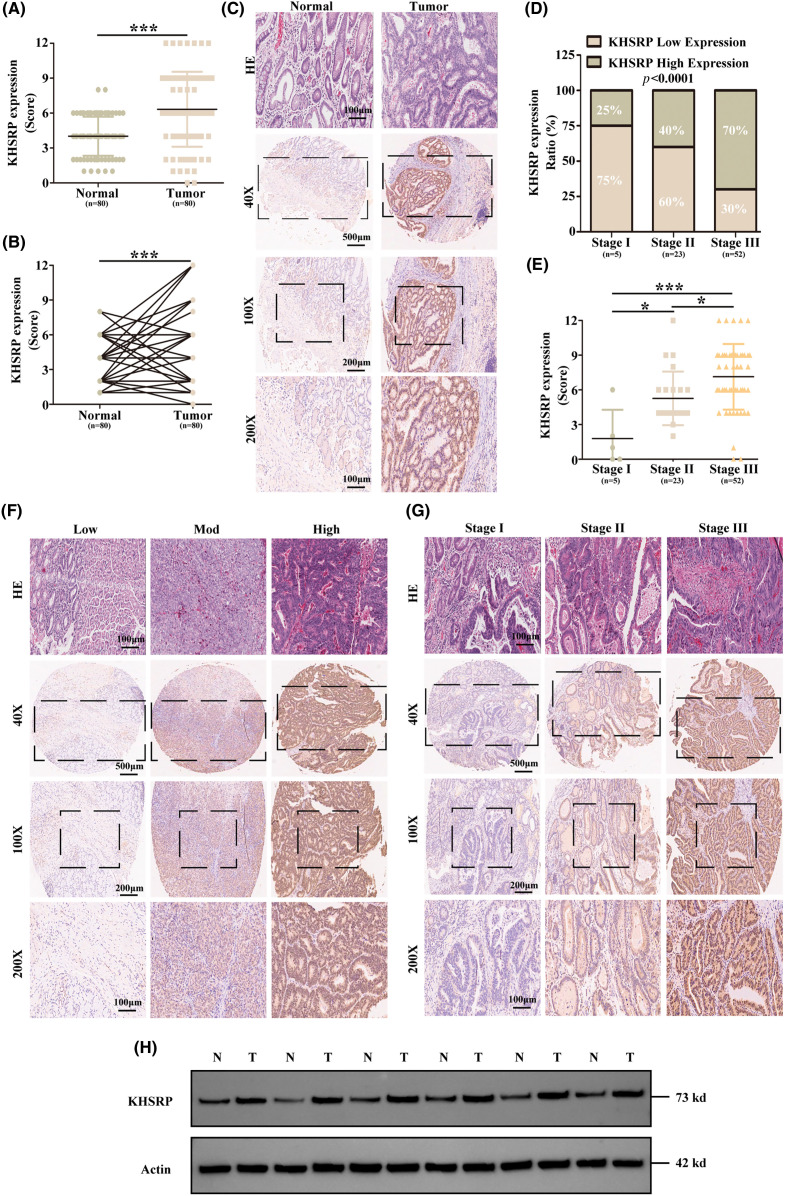
KHSRP is significantly upregulated in gastric cancer tissues and is highly correlated with the malignancy of the tumor. (A and B) IHC scoring was used to quantitatively assess KHSRP expression in 80 gastric cancer tissues and 80 adjacent non-cancerous tissues. (C) Representative images of KHSRP expression in non-cancerous and gastric cancer tissues. (D) IHC score analysis and corresponding statistics for KHSRP expression in gastric cancer tissues across different TNM stages. (E) Statistical comparison of KHSRP expression levels across various TNM stages. (F) Representative images showing low, medium, and high KHSRP expression levels in gastric cancer tissues, as detected by immunohistochemistry. Scale bar: 100 μm. (G) Representative images of KHSRP expression in gastric cancer tissues at different TNM stages, identified via immunohistochemistry. Scale bar: 100 μm. (H) Western blot analysis comparing KHSRP protein expression in non-cancerous and gastric cancer tissues. *p < 0.05, ***p < 0.001.

To verify the expression level of KHSRP protein in gastric cancer tissues, we performed immunohistochemical analysis using gastric cancer tissue microarrays (TMAs). The results demonstrated that KHSRP expression was significantly higher in gastric cancer tissues compared to normal gastric mucosa, as indicated by elevated staining scores (Fig. 2A–C). Furthermore, increased KHSRP expression was associated with poorer histological differentiation and more advanced clinical stages of gastric cancer (Fig. 2D–G). In addition, we collected clinical samples and conducted Western blot analysis to confirm KHSRP expression in gastric cancer and adjacent non-cancerous tissues. The results showed that KHSRP expression was elevated in gastric cancer tissues compared to adjacent non-cancerous tissues (Fig. 2H). These findings indicate that KHSRP is upregulated in gastric cancer tissues and that its expression level is positively correlated with tumor malignancy.

### KHSRP–GOF enhances CSC–like properties, tumor growth and metastasis

**Figure 3:**
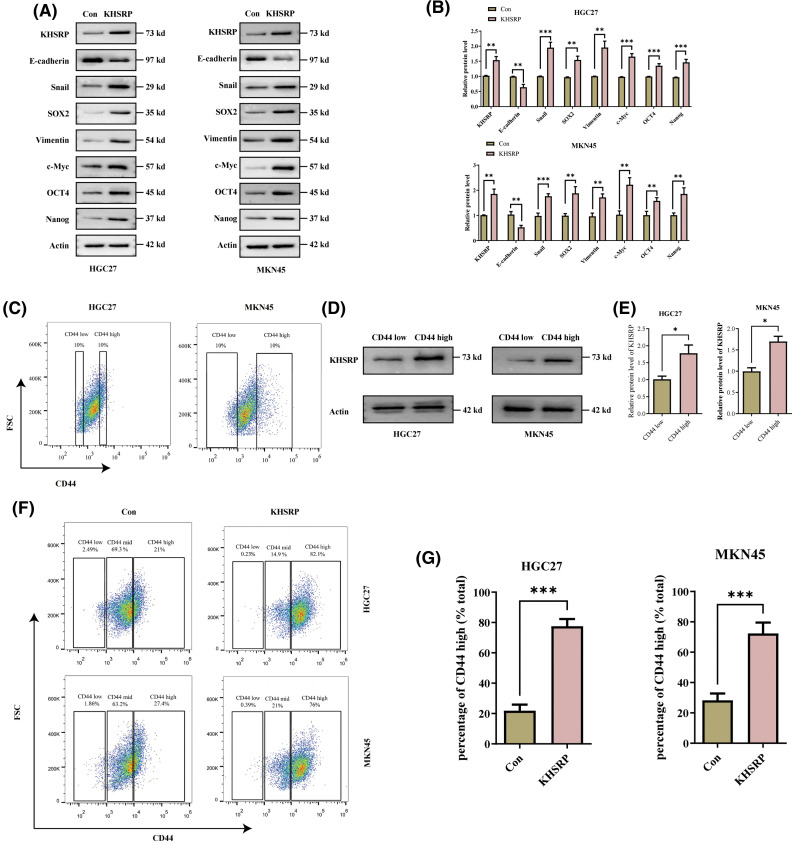

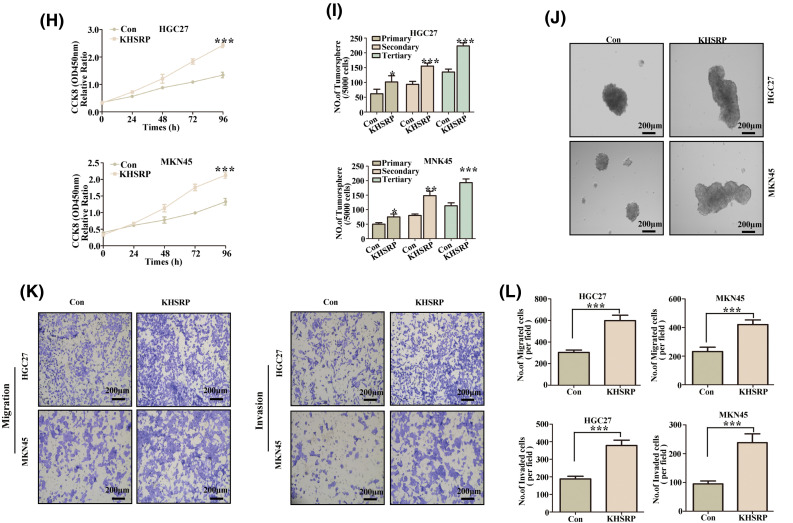
KHSRP–GOF enhances CSC–like properties, tumor growth and metastasis. (A and B) Western blot analysis was used to examine the expression of EMT-related and cancer stem cell-associated proteins in gastric cancer cells with stable expression of either control (Con) or overexpressed KHSRP. (C) Fluorescence-activated cell sorting was used to isolate gastric cancer cells with high and low expression of CD44. (D and E) Expression of KHSRP protein in CD44 high-expression and low-expression groups was detected by Western blot analysis. (F and G) Flow cytometry was used to assess the expression of CD44 in KHSRP overexpression and control groups. (H) The CCK-8 assay evaluated the effect of stable KHSRP overexpression on gastric cancer cell proliferation by measuring cell viability. (I and J) Tumorsphere formation assays were conducted to assess the self-renewal capacity of cancer stem cells following KHSRP overexpression, with representative images of tumorspheres shown. (K and L) Transwell assays were performed to evaluate the migration and invasion capabilities of gastric cancer cells with stable KHSRP overexpression *in vitro*. The left panel presents representative Transwell images, while the right panel provides the statistical analysis. *p < 0.05, **p < 0.01, ***p < 0.001.

To elucidate the role of KHSRP in gastric cancer, we generated HGC27 and MKN-45 cell lines with overexpressed KHSRP. Western blot and quantitative polymerase chain reaction (qPCR) analysis confirmed successful overexpression of KHSRP in these cells (Fig. 3A–B and Fig. S1A). We then assessed the impact of KHSRP overexpression on cell proliferation using CCK8 assays, which revealed a significant promotion of cell proliferation in gastric cancer cells (Fig. 3H). To investigate the tumor stemness in apple tumor cells, we used flow cytometric sorting technology to isolate tumor cells with high and low expression of CD44. Western blot analysis revealed that the expression of KHSRP protein was significantly higher in the CD44 high-expression group compared to the CD44 low-expression group (Fig. 3C–E). Furthermore, flow cytometric analysis showed that the CD44 expression in the KHSRP overexpression group was significantly higher than that in the control group (Fig. 3F–G). We evaluated the effect of KHSRP on tumor stem cell characteristics through tumor sphere formation assays. The results showed that KHSRP markedly enhanced the self-renewal capacity of gastric cancer stem cells (Fig. 3I–J). Additionally, Transwell assays demonstrated that KHSRP overexpression significantly increased cell migration and invasion (Fig. 3K–L). Further Western blot analysis revealed that KHSRP overexpression downregulated E-cadherin while upregulating key mesenchymal markers, including N-cadherin, vimentin, and Snail (Fig. 3A–B). Moreover, the expression of stemness-related proteins such as SOX2, OCT4, Nanog, and c-Myc was also significantly elevated (Fig. 3A–B). These findings suggest that KHSRP overexpression enhances the stemness, proliferation, and migration of gastric cancer cells, underscoring its oncogenic potential in gastric cancer.

### KHSRP–LOF reduces CSC–like properties, tumor growth and metastasis *in vitro*

**Figure 4:**
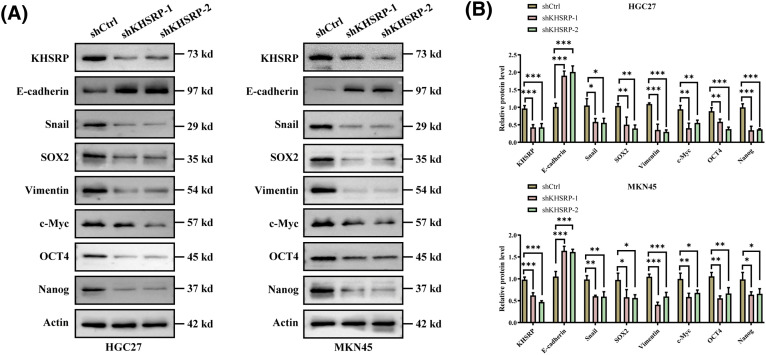

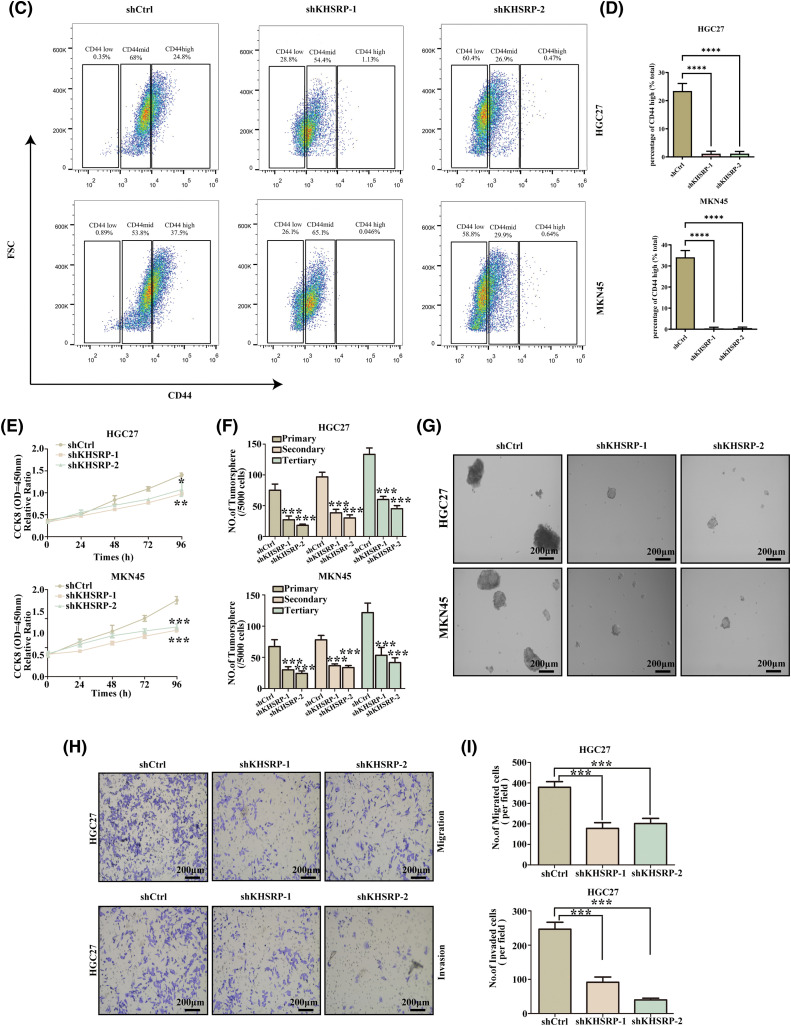
KHSRP–LOF reduces CSC–like properties, tumor growth and metastasis *in vitro*. (A and B) Western blot analysis was conducted to examine the levels of EMT-related and cancer stem cell-associated proteins in gastric cancer cells with stable expression of shCtrl or shKHSRP. (C and D) Flow cytometry was used to assess the expression of CD44 in KHSRP knockdown and control groups. (E) The CCK-8 assay measured cell viability to assess the impact of stable shCtrl or shKHSRP expression on gastric cancer cell proliferation. (F and G) Tumorsphere formation assays were used to evaluate the self-renewal ability of cancer stem cells after stable expression of shCtrl or shKHSRP in gastric cancer cells, with representative images provided. (H and I) Transwell assays assessed the effects of stable shCtrl or shKHSRP expression on the migration and invasion capabilities of gastric cancer cells *in vitro*. *p < 0.05, **p < 0.01, ***p < 0.001, ****p < 0.0001.

To further elucidate the role of KHSRP in gastric cancer cells, we used lentiviral vectors to transduce three different shRNAs (shKHSRP-1, shKHSRP-2, and shKHSRP-3) into HGC27 and MKN-45 cells. qPCR analysis indicated that shKHSRP-1 and shKHSRP-2 had good transfection efficiency (Fig. S1A). Therefore, we selected shKHSRP-1 and shKHSRP-2 for Western blot validation of transfection efficiency, and the results showed that shKHSRP-1 and shKHSRP-2 significantly reduced the expression of KHSRP protein (Fig. 4A–B and Fig. S1A). We then investigated the effects of KHSRP knockdown on cell proliferation, tumor stem cell self-renewal, and the invasion and migration capabilities of gastric cancer cells. The results contrasted sharply with those observed in KHSRP overexpression experiments. Furthermore, flow cytometric analysis showed that the CD44 expression in the KHSRP knockdown group was significantly lower than that in the control group (Fig. 4C–D). The CCK8 assay revealed that KHSRP knockdown significantly inhibited the viability and proliferation of gastric cancer cells (Fig. 4E). Moreover, the tumor sphere formation assay showed that the downregulation of KHSRP markedly reduced both tumor-sphere formation ability and stemness (Fig. 4F–G). Transwell assays further confirmed that KHSRP knockdown significantly suppressed the migration and invasion capacities of gastric cancer cells (Fig. 4H–I and Fig. S1C,D). Western blot analysis demonstrated that KHSRP downregulation led to an increase in E-cadherin expression (an epithelial marker) while reducing the expression of vimentin, Snail (mesenchymal markers), and stemness-related proteins (Fig. 4A–B). These findings indicate that KHSRP inhibition significantly impairs the self-renewal capacity, proliferation, and migration abilities of gastric cancer stem cells.

### KHSRP enhances CSC-like properties, tumor growth, and metastasis via the WNT pathway

**Figure 5:**
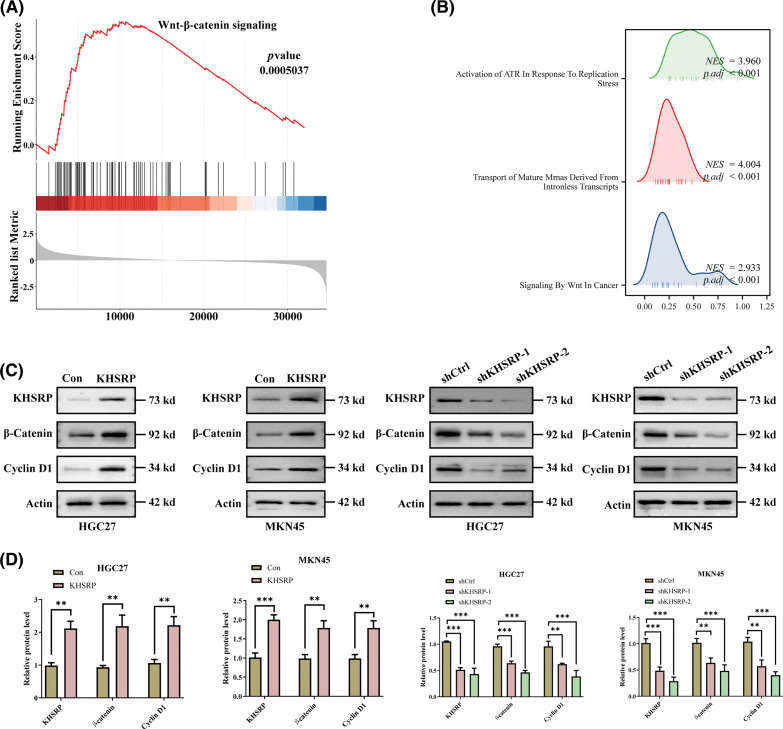

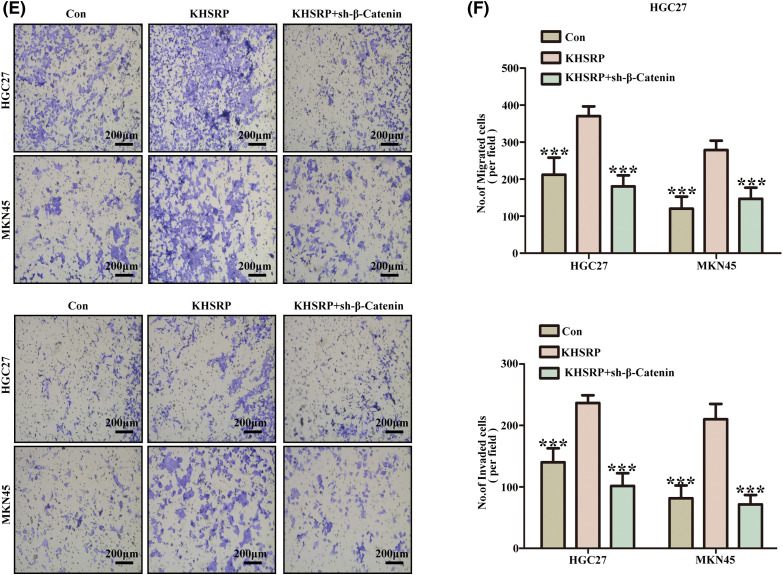
KHSRP Enhances CSC-Like properties, Tumor Growth, and Metastasis via the WNT Pathway. (A and B) Gene set enrichment analysis. (C and D) Western blot analysis of KHSRP, β-catenin, and Cyclin D1 expression in HGC27 and MKN45 gastric cancer cells with either KHSRP overexpression or knockdown. Actin was used as a loading control. (E and F) Transwell migration and invasion assays were performed in HGC27 and MKN45 cells to assess the effects of KHSRP overexpression or KHSRP knockdown combined with β-catenin knockdown. **p < 0.01, ***p < 0.001.

TCGA data analysis revealed that KHSRP expression is associated with the activation of pathways including Activation of ATR In Response To Replication, Transport of Mature mRNAs Derived From Intronless Transcripts, and Signaling by Wnt in Cancer (Fig. 5A–B). Further Western blot analysis demonstrated that KHSRP overexpression increased β-catenin and Cyclin D1 protein levels in both HGC27 and MKN45 cells. Conversely, silencing KHSRP reduced the expression of β-catenin and Cyclin D1 (Fig. 5C–D). Additionally, silencing β-catenin reversed the enhanced migratory ability induced by KHSRP overexpression (Fig. 5E–F).

### Silencing KHSRP enhances anti-tumor immunity in C3H/He mice

**Figure 6:**
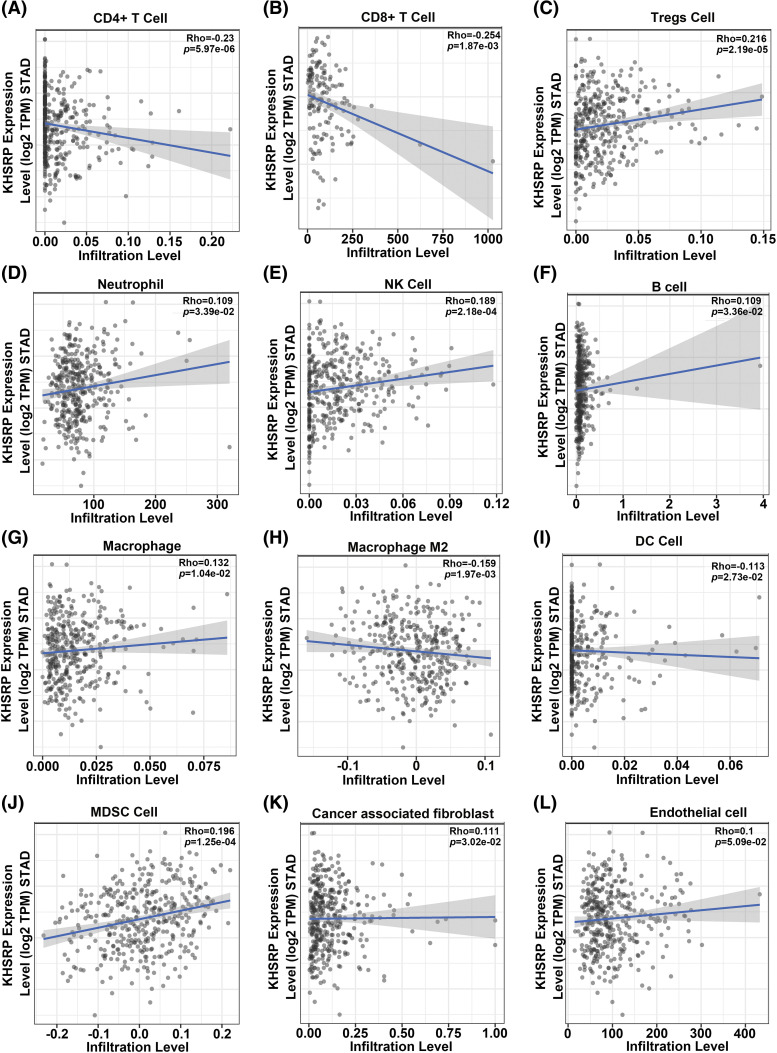
Correlation between KHSRP expression and immune cell infiltration levels in gastric cancer (STAD) based on TCGA data. (A and B) Negative correlation between KHSRP expression and infiltration levels of CD4+ T cells (A) and CD8+ T cells (B). (C) Negative correlation between KHSRP expression and regulatory T cells (Tregs) infiltration. (D–F) Positive correlation of KHSRP expression with infiltration levels of neutrophils (D), NK cells (E), and B cells (F). (G and H) KHSRP expression shows a positive correlation with macrophages (G) and M2 macrophages (H). (I–L) KHSRP expression correlates positively with infiltration levels of dendritic cells (I), myeloid-derived suppressor cells (MDSCs) (J), cancer-associated fibroblasts (K), and endothelial cells (L). Data are presented as scatter plots, with the line of best fit showing the correlation trend. Rho and *p*-values are displayed for each comparison.

**Figure 7:**
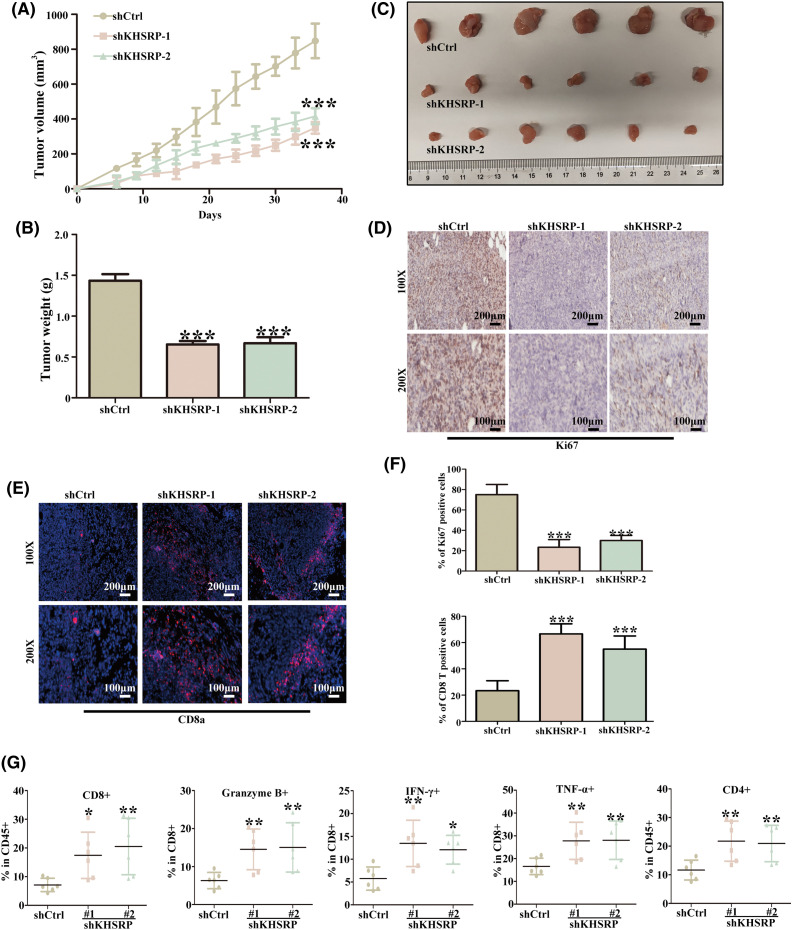
Silencing KHSRP Enhances Anti-Tumor Immunity in C3H/He Mice. (A) Tumor volume growth curves in mice with subcutaneous xenografts of shCtrl, shKHSRP-1, and shKHSRP-2 HGC27 cells. ***p < 0.001. (B) Tumor weight at the endpoint of the experiment (Day 40) showed a significant reduction in the KHSRP knockdown groups compared to the control group. ***p < 0.001. (C) Representative images of the excised tumors from mice in each group, with scale provided. (D) Immunohistochemistry (IHC) staining for Ki67, showed reduced proliferation in tumors with KHSRP knockdown. (E) Immunofluorescence staining for CD8α+ T cells, showed increased infiltration of CD8α+ cells in the KHSRP knockdown tumors. (F) Quantification of CD8α+ T cells from immunofluorescence staining. ***p < 0.001. (G) Flow cytometry analysis showed an increase in CD4+, CD8+, Granzyme B+, IFN-γ+, and TNF-α+ immune cells in the KHSRP knockdown tumors compared to controls. Data are presented as mean ± SD, with *p < 0.05, **p < 0.01.

Analysis of TCGA data revealed a strong negative correlation between KHSRP expression and both CD4+ and CD8+ T cell infiltration (Fig. 6A–L). In a mouse xenograft model, we further observed a significant reduction in tumor volume in the KHSRP-silenced group (Fig. 7A–C). Immunohistochemical staining revealed a notable reduction in Ki67 expression, while immunofluorescence indicated a substantial increase in CD8α+ cell infiltration in the KHSRP-silenced group (Fig. 7D–F). Additionally, flow cytometry analysis demonstrated a significant increase in CD4+, CD8+, Granzyme B+, IFN-γ+, and TNF-α+ cells in the KHSRP-silenced group (Fig. 7G and Fig. S1E).

## Discussion

GC remains one of the most lethal cancers worldwide, with most cases diagnosed at an advanced stage when treatment options are limited, resulting in a poor prognosis. Despite the availability of conventional treatments such as surgery and chemotherapy, the high mortality rate associated with GC continues to present significant challenges [38]. Early detection, however, can significantly improve treatment outcomes, enabling the use of more effective strategies, including molecular targeted therapies. For instance, targeting HER2 and VEGFR with drugs like trastuzumab and apatinib has offered survival benefits for certain patients [39,40]. However, effective therapeutic targets and drugs for GC remain limited in clinical practice, highlighting the urgent need for the discovery of new biological targets. Additionally, research has identified a rare subpopulation of cancer cells known as CSCs, which possess strong tumorigenic potential and play a critical role in sustaining the malignancy [41,42]. This study highlights the role of KHSRP in enhancing the self-renewal capacity of gastric cancer stem cells while weakening anti-tumor immunity, thereby driving tumor progression and positioning KHSRP as a potential prognostic marker for gastric cancer patients.

Tumors exhibit significant heterogeneity, largely driven by a subpopulation of CSCs that can initiate and sustain tumor growth while resisting treatment. These cells are characterized by their ability to self-renew and undergo asymmetric division, giving rise to both new CSCs and more differentiated cells, which contribute to tumor heterogeneity. Previous studies have identified cluster of differentiation 44 (CD44) and aldehyde dehydrogenase (ALDH) activity as key markers of the tumorigenic and drug-resistant CSC subpopulations in gastric cancer. These CSCs constitute approximately 0.5% to 3.5% of primary gastric cancer cells in patients [43]. The high mortality rate in gastric cancer is closely associated with drug resistance and tumor recurrence after treatment, highlighting the critical role that CSCs play in these outcomes [44]. Current research is focused on developing strategies to target the self-renewal capabilities of gastric CSCs, either by inducing their differentiation or by disrupting their metabolism [45,46]. Consequently, targeting specific CSC signaling pathways could offer new therapeutic avenues. Our study identifies KHSRP as a potential marker for gastric cancer stem cells and suggests that targeting KHSRP may help eliminate these CSCs, thereby inhibiting the malignant progression of gastric cancer.

KHSRP is a multifunctional nucleic acid-binding protein that belongs to the far upstream element-binding protein (FUBP) family, which also includes FUBP1, FUBP2, and FUBP3 [29,32]. This family is known for its ability to bind single-stranded DNA. KHSRP plays a crucial role in various RNA-related processes, including splicing, transport, editing, as well as mRNA stability and degradation [47]. KHSRP is also implicated in several cellular processes associated with neuromuscular diseases, obesity, type II diabetes, and cancer [31].

Several studies have investigated KHSRP’s role in tumorigenesis and cancer progression, revealing its diverse functions across different tumor types. For instance, in human glioma cells, KHSRP has been shown to have no significant impact on cell proliferation; however, its low expression can promote tumor formation [48]. In non-small cell lung cancer (NSCLC), higher levels of KHSRP are associated with longer patient survival and reduced cell migration and metastasis, suggesting that KHSRP may play a tumor-suppressive role in this context [35]. However, other studies have shown that silencing KHSRP reduces cell proliferation, reverses anchorage-independent growth, and decreases migration and invasion, indicating a potential oncogenic role for KHSRP in lung cancer [49,50]. In contrast, in liver cancer, KHSRP has been found to have varying effects. FUBP1 and FUBP2 have been shown to promote proliferation and invasion, while another study observed that these proteins are highly expressed in less differentiated liver cancer tissues [51,52].

Reports on the role of KHSRP in gastric cancer are limited, particularly regarding its involvement in cancer stem cell maintenance, metastasis, and anti-tumor immunity. KHSRP is highly expressed in gastric cancer tissues and is closely associated with malignant progression. It plays a critical role in maintaining the stemness of gastric cancer stem cells and promoting their metastatic potential. *In vitro* experiments demonstrated that KHSRP silencing significantly inhibited the migration and invasion of gastric cancer cells, likely by disrupting their stemness. Additionally, KHSRP expression was elevated in gastric cancer tissues compared to adjacent normal tissues and was significantly correlated with tumor stage, lymph node metastasis, and distant metastasis. *In vivo* experiments further revealed that KHSRP silencing markedly increased the infiltration of CD4+, CD8+, Granzyme B+, IFN-γ+, and TNF-α+ cells within the tumor, suggesting that KHSRP could serve as a potential prognostic marker and therapeutic target for gastric cancer.

The current study is limited to the functional analysis of the KHSRP gene, and its specific mechanisms and target effects remain unknown, necessitating further exploration. Additionally, achieving complete knockout of this gene would yield more reliable results.

## Conclusion

KHSRP acts as an oncogene in gastric cancer by promoting tumorigenesis and suppressing anti-tumor immune responses. Its overexpression is associated with poor prognosis, making KHSRP a potential prognostic marker and therapeutic target in gastric cancer.

## Supplementary Materials

Figure S1 (A) Quantitative PCR (qPCR) analysis showing the relative expression levels of the indicated genes. Data are presented as mean ± SEM from three independent experiments. Statistical significance was determined using Student's t-test, ns: p≥0.05, **p< 0.01, ***p< 0.001, ****p< 0.0001. (B) Expression levels of the KHSRP gene in different pathological grades of gastric cancer. Data are presented as mean ± SEM. Statistical comparisons between groups were performed using one-way ANOVA. (C) Representative images of Transwell migration and invasion assays. Migrated and invaded cells were stained and imaged under a light microscope. (D) Quantitative analysis of Transwell migration and invasion experiments. The number of migrated and invaded cells was quantified from three independent experiments and presented as mean ± SEM. ***p< 0.001.(E) Flow cytometry gating strategy for cell sorting. Gated populations represent the subsets used in downstream analysis.

## Data Availability

The datasets used in this study are available in the GEPIA database and the TCGA database.

## Abbreviations

ANOVA: Analysis of Variance
AREs: AU-Rich Elements
BRCA: Breast Carcinoma/Breast Invasive Carcinoma
CCK8: Cell Counting Kit-8
CD4+: Cluster of Differentiation 4 Positive
CD8+: Cluster of Differentiation 8 Positive
CESE: Cervical Squamous Cell Carcinoma and Endocervical Adenocarcinoma
CHOL: Cholangiocarcinoma
COAD: Colon Adenocarcinoma
ECL: Enhanced Chemiluminescence
EMT: Epithelial-Mesenchymal Transition
ESCA: Esophageal Carcinoma
FBS: Fetal Bovine Serum
FUBP: Far Upstream Element-Binding Protein
GBM: Glioblastoma Multiforme
GC: Gastric Cancer
GEPIA: Gene Expression Profiling Interactive Analysis
GOF: Gain-of-function
HNSC: Head and Neck Squamous Cell Carcinoma
IHC: Immunohistochemistry
KIRC: Kidney Renal Clear Cell Carcinoma
KIRP: Kidney Renal Papillary Cell Carcinoma
LIHC: Liver Hepatocellular Carcinoma
LUAD: Lung Adenocarcinoma
LUSC: Lung Squamous Cell Carcinoma
MDSCs: Myeloid-Derived Suppressor Cells
NK: Natural Killer
NS: Not Significant
OS: Overall Survival
PBS: Phosphate-buffered Saline
PCPG: Pheochromocytoma and Paraganglioma
PRAD: Prostate Adenocarcinoma
RIPA: Radio-Immunoprecipitation Assay
RPMI: Roswell Park Memorial Institute
SARC: Sarcoma
SKCM: Skin Cutaneous Melanoma
SPF: Specific Pathogen-Free
STAD: Stomach Adenocarcinoma
SYSU-IACUC: Sun Yat-sen University Institutional Animal Care and Use Committee
THYM: Thymoma
TMAs: Tissue Microarrays
UCEC: Uterine Corpus Endometrial Carcinoma
UTRs: Untranslated Regions

## Supplementary Materials

The supplementary material is available online at https://doi.org/10.32604/or.2024.058273.
