# Supplementary figures and images for: KHSRP promotes cancer stem cell maintenance, tumorigenesis, and suppresses anti-tumor immunity in gastric cancer
Source: Oncol Res. 2025 Jan 16;33(2):309–25. doi: 10.32604/or.2024.058273 (PMC11753988; doi:10.32604/or.2024.058273)

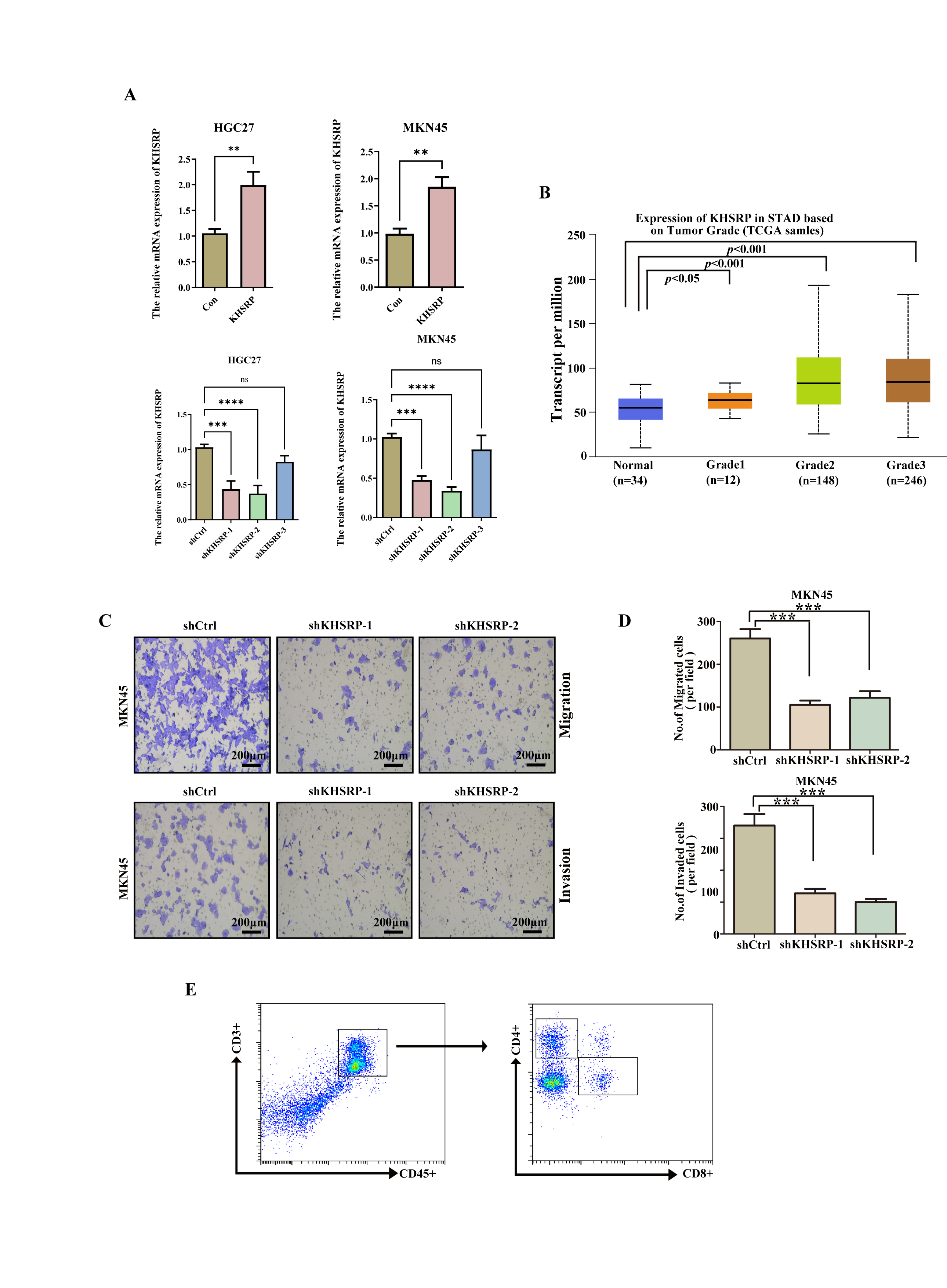

Supplement: Figure S1 [file OncolRes-33-58273-s001.tif]
