# Supplementary material for: KHSRP promotes cancer stem cell maintenance, tumorigenesis, and suppresses anti-tumor immunity in gastric cancer
Source: Oncol Res. 2025 Jan 16;33(2):309–25. doi: 10.32604/or.2024.058273 (PMC11753988; doi:10.32604/or.2024.058273)
Supplement: Supplementary file 2 [file OncolRes-33-58273-s002.docx]

**Table S1: List of antibodies used in this study**

| **Target protein** | **Description** | **Company**  **Cat no.** | **Dilution ratio** | **Experiment used** |
| --- | --- | --- | --- | --- |
| KHSRP | KHSRP Polyclonal antibody | Proteintech  55409-1-AP | 1:1000 | WB/IF |
| β-catenin | Beta Catenin Monoclonal antibody | Proteintech  66379-1-Ig | 1:5000 | WB |
| c-Myc | c-MYC Monoclonal antibody | Proteintech  67447-1-Ig | 1:5000 | WB |
| Cyclin D1 | Cyclin D1 Monoclonal antibody | Proteintech  60186-1-Ig | 1:5000 | WB |
| β-actin | Mouse anti DDDDK-Tag monoclonal antibody | ABclonal  AE005 | 1:20000 | WB |
| N-cadherin | N-cadherin Monoclonal antibody | Proteintech  66219-1-Ig | 1:5000 | WB |
| E-cadherin | E-cadherin Monoclonal antibody | Proteintech  60335-1-Ig | 1:2000 | WB |
| Vimentin | Vimentin Monoclonal antibody | Proteintech  60330-1-Ig | 1:20000 | WB |
| OCT4 | OCT4 Monoclonal antibody | Proteintech  60242-1-Ig | 1:5000 | WB |
| Nanog | NANOG Monoclonal antibody | Proteintech  67255-1-Ig | 1:5000 | WB |
| Snail | Snail Rabbit pAb | Abclonal A5243 | 1:500 | WB |
| Anti-Rabbit | HRP goat anti-rabbit IgG (H+L) | ABclonal  AS014 | 1:2000 | WB |
| Anti-Mouse | HRP goat anti-mouse IgG (H+L) | ABclonal  AS003 | 1:2000 | WB |
| CD44 | CD44 PE anti-human/mouse | ThermoFisher  12-0441-82 | 0.125 µg/ test | Flow  cytometry |
| CD45 | CD45 Brilliant Violet 605 anti-mouse | BioLegend  103140 | 1:20 | Flow  cytometry |
| CD3 | CD3 PE anti-mouse | BioLegend  100308 | 0.25µg/ test | Flow  cytometry |
| CD4 | CD4 FITC anti-mouse | BioLegend  100510 | 0.25µg/ test | Flow  cytometry |
| CD8 | CD8 Brilliant Violet 421 anti-mouse | BioLegend  100738 | 1:20 | Flow  cytometry |
| Granzyme B | Granzyme B PerCP/Cy5.5 anti-mouse | BioLegend  372212 | 1:20 | Flow  cytometry |
| TNF-α | TNF-α PE/Cy7 anti-mouse | BioLegend  506324 | 0.25µg/ test | Flow  cytometry |
| IFN-γ | IFN-γ FITC anti-mouse | BioLegend  505806 | 1.0µg/ test | Flow  cytometry |
| CD8a | CD8A Rabbit mAb | ABclonal  A23081 | 1:100 | IF |
| anti-mouse | Alexa Fluor® 594 anti-mouse | Invitrogen  A21203 | 1:500 | IF |
| anti-rabbit | Alexa Fluor® 488 anti-rabbit | Invitrogen  A27034 | 1:500 | IF |
| Ki67 | Ki67 Rabbit mAb | ABclonal A20018 | 1:200 | IHC |
